# Genome-wide association study of Stevens-Johnson Syndrome and Toxic Epidermal Necrolysis in Europe

**DOI:** 10.1186/1750-1172-6-52

**Published:** 2011-07-29

**Authors:** Emmanuelle Génin, Martin Schumacher, Jean-Claude Roujeau, Luigi Naldi, Yvonne Liss, Rémi Kazma, Peggy Sekula, Alain Hovnanian, Maja Mockenhaupt

**Affiliations:** 1 Inserm U946, F-75010, Paris, France; 2 Institut Universitaire d'Hématologie, Université Paris Diderot, F-75010, Paris, France; 3 Institute of Medical Biometry and Medical Informatics, University Medical Center, D-79095Freiburg, Germany; 4 Inserm U448, F-94010, Créteil, France; 5 Service Dermatologie, Hôpital Henri-Mondor, Université Paris-Est, F-94010, Créteil, France; 6 Department of Dermatology, Azienda Ospedaleria Ospedali Riuniti di Bergamo, Milano University, Bergamo, Italy; 7 Dokumentationszentrum schwerer Hautreaktionen (dZh), Department of Dermatology, D-79095, Freiburg, Germany; 8 INSERM U781, F-75743, Paris, France; 9 Université René Descartes, F-75743, Paris, France; 10 Centre Hospitalier Universitaire Necker-Enfants malades, Departments of Genetics and Dermatology, F-75743, Paris, France

## Abstract

**Background:**

Stevens-Johnson syndrome (SJS) and Toxic Epidermal Necrolysis (TEN) are rare but extremely severe cutaneous adverse drug reactions in which drug-specific associations with HLA-B alleles were described.

**Objectives:**

To investigate genetic association at a genome-wide level on a large sample of SJS/TEN patients.

**Methods:**

We performed a genome wide association study on a sample of 424 European cases and 1,881 controls selected from a Reference Control Panel.

**Results:**

Six SNPs located in the HLA region showed significant evidence for association (OR range: 1.53-1.74). The haplotype formed by their risk allele was more associated with the disease than any of the single SNPs and was even much stronger in patients exposed to allopurinol (OR_allopurinol _= 7.77, 95%CI = [4.66; 12.98]). The associated haplotype is in linkage disequilibrium with the HLA-B*5801 allele known to be associated with allopurinol induced SJS/TEN in Asian populations.

**Conclusion:**

The involvement of genetic variants located in the HLA region in SJS/TEN is confirmed in European samples, but no other locus reaches genome-wide statistical significance in this sample that is also the largest one collected so far. If some loci outside HLA play a role in SJS/TEN, their effect is thus likely to be very small.

## Background

Adverse drug reactions are a major public health issue as they represent an important cause of morbidity and mortality [1]. Skin lesions are frequent expressions of adverse drug reactions with, for some drugs, up to 10% of cutaneous reactions observed [2,3]. SJS and TEN are severe cutaneous adverse reactions characterized by the development of acute exanthema which progresses towards limited (in SJS) or widespread (in TEN) blistering and erosion of the skin and mucous membranes [4]. SJS and TEN are thus considered to be two different forms of the same disease with TEN representing the most severe form [5]. The incidence of SJS/TEN is estimated to be of approximately 1-2 patients per million individuals per year [6]. It is thus a very rare disease but with a high morbidity and mortality (reaching up to 45% in TEN) that requires intensive treatment.

SJS/TEN is not associated with a single drug or a single group of drugs but several different drugs have been involved. A limited number of drugs however are more often associated with the disease: antibacterial sulfonamides (especially sulfamethoxazole), allopurinol, which is the most frequent drug involved [7], carbamazepine, lamotrigine, phenobarbital, phenytoine, non-steroidal anti-inflammatory drugs (NSAIDs) of the oxicam type and neviparine [4,8]. Only a small number of individuals exposed to these "highly suspected" drugs develop the disease and a genetic susceptibility has been suggested [9-12].

An association with HLA was reported more than 20 years ago [12,13]. More recently, studies in the Han Chinese population have involved the HLA-B locus with very strong drug-specific associations: the HLA-B*1502 allele was found in all carbamazepine-induced SJS/TEN patients [14] and the HLA-B*5801 in all allopurinol-induced SJS/TEN patients [15]. An investigation of HLA-B associations in European samples did not detect the association between the HLA-B*1502 allele and carbamazepine-induced SJS/TEN but did report a strong but not complete association between HLA-B*5801 and the allopurinol-induced disease [16,17]. Furthermore, it has also been suggested that HLA genetic predisposition may not be the same for SJS and TEN and that it might thus be important to take into account the disease severity in association tests [18].

Apart from HLA, several other candidate genes have been tested for association with SJS/TEN, mostly genes involved in the immune response, in the inflammation process or in drug metabolism [18-21]. However, no association with these genes has been consistently found and it has been suggested that rather than focusing on candidate genes, a genome-wide association study might provide more insights into the genetic susceptibility of adverse drug reactions [21].

In the context of the RegiSCAR project (European Registry of Severe Cutaneous Adverse Reactions to Drugs and Collection of Biological Samples) DNA of 563 cases of SJS/TEN was collected. This is the largest available sample of SJS/TEN patients in the world with accurate medical information regarding the severity of the disease and the history of drug intake. These patients were genotyped at the Centre National de Genotypage (CNG) using Illumina 317 K chips and a Genome Wide Association Study (GWAS) was conducted against controls selected from the CNG European Reference Control Panel [22].

## Methods

### Patients

A total of 563 cases (226 males and 337 females, sex-ratio = 0.67) were included in this study. They were collected as part of the "European Registry of Severe Cutaneous Adverse Reactions" (RegiSCAR) (see http://regiscar.uni-freiburg.de/) in six countries (Austria (1 case), France (184 cases), Germany (331 cases), Israel (14), Italy (26) and The Netherlands (7)). All of them had a diagnosis of SJS (268 cases, 48%), SJS/TEN overlap (181 cases, 32%) or TEN (114 cases, 20%) validated as probable or definitive by the expert committee of RegiSCAR blindly from information on drug exposure.

For each patient, written informed consent was obtained and a blood sample was taken for genomic DNA extraction that was carried out at the CEPH-Fondation Jean Dausset (France) (http://www.cephb.fr) until 2005 and at the biobank of the CIC-Henri Mondor / Créteil after 2005.

The 563 patients were genotyped on Illumina 317 K chips in France at the CNG (http://www.cng.fr) for a total of 318,127 SNPs (among which 309,091 were located on autosomes). Stringent quality-control (QC) was performed using Plink v1.06 [23] that led to the exclusion of 68 out of the 563 cases for low genotyping (MIND > 0.05) and 15,088 SNPs (14,343 autosomal SNPs) for missing data (GENO > 0.05). After QC, a total of 495 affected individuals were considered in the analysis for whom genotypes on 303,039 SNPs (294,748 located on autosomes) were available. The total genotyping rate in these 495 remaining individuals was 0.95.

### Controls

Controls were selected from the CNG European Reference Panel collected at the CNG [22]. This panel includes 5,847 unrelated individuals from 13 European countries who are all genotyped on Illumina 317 K chips. Since patients collected as part of the RegiSCAR project were mostly from France and Germany, we decided to consider only the 1,881 individuals from the Reference Control panel originating from these two countries (653 from Germany and 1,228 from France).

### Quality control and principal component analysis of genotype data

A second quality control of the data was performed where markers that were missing in more than 5% of the controls or had significant different missing rates between cases and controls were removed. A total of 25,834 autosomal markers were excluded.

**Figure 1:**
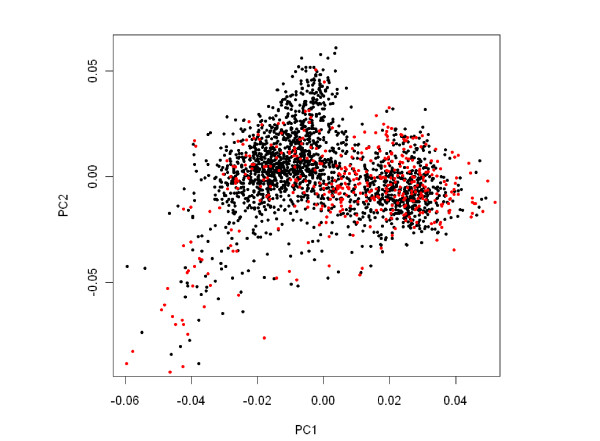
**Plots of top two principal components for cases (in red) and controls (in black)**.

To assess the level of population stratification in the sample, a principal component analysis (PCA) was performed using the genotypes of the 495 cases and 1,881 controls for a sub-panel of 35,232 markers obtained after pruning based on linkage disequilibrium using Plink (option indep-pairwise 50 5 0.1). The program smartPCA from the Eigenstrat package [24,25] was used with the default options. A total of 71 patients were excluded from the study as they were found to be outliers. Those were mostly individuals with a suspected African or Asian ancestry. Plots of the first two principal components (PCs) are provided in Figure 1.

**Figure 2:**
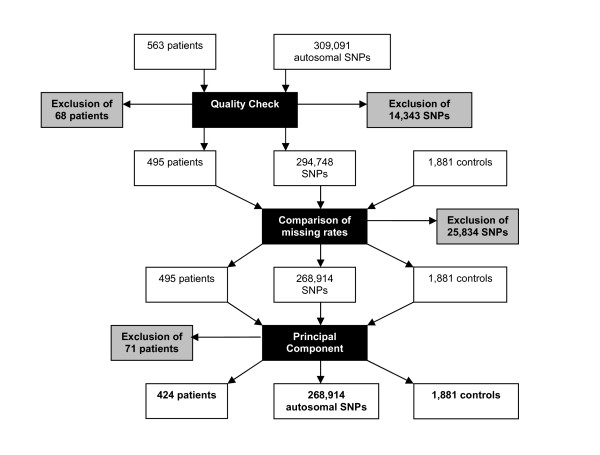
**Flowchart summarizing the different steps of the quality control of the data**.

As shown in the flowchart in Figure 2, a total of 268,914 autosomal SNPs were kept for the association testing in a sample of 424 SJS/TEN patients and 1,881 controls.

### Drug exposure

Information on drug exposure was collected by interviewing patients about their drug intakes and by consulting medical records. Reactions were considered to be potentially caused by allopurinol, carbamazepine or phenytoin if the drug has been taken between 4 and 10 days before the onset of the disease and if the drug has not been started more than 42 days before onset. This resulted in a group of 57 patients (13.4%) with potentially allopurinol-induced reactions, a group of 25 patients (5.9%) with potentially carbamazepine-induced reactions and a group of 19 patients (4.5%) with potentially phenytoin-induced reactions. Because of the low number in the latter two groups we decided to only compare the allopurinol group and the group with other drugs to the controls in this study. Note that because of the stringent time criterion used to determine allopurinol as culprit drug there might be some patients with an allopurinol-induced reaction left in the group of patients with a culprit drug other than allopurinol. For this reason, this analysis was further enforced by an additional analysis where any allopurinol exposed patient (irrelevant of time of usage) was assigned to the allopurinol group resulting in a higher sensitivity with respect to allopurinol-induced reaction while the specificity is decreased.

### Association testing

Association was tested using the PC-corrected Armitage-trend test implemented in Eigenstrat [24]. A sensitivity analysis was performed to determine the number of PCs that should be accounted for and we decided to adjust only on the first two PCs (the genomic control coefficient lambda [26] was 1.023 when adjusting on the top 2 PCs, 1.019 when adjusting on the top 10 PCs and 1.016 when adjusting on the top 20).

Odds-ratios (OR) and confidence intervals of the different genotypes were computed using the logistic regression model implemented in Plink v1.06 [23].

To study the influence of the drug involved in the disease, a second study was performed where the multinomial logistic model implemented in Stata v10 [27] was used as in [28]. The disease status D was coded in 3 classes: 0 unaffected (controls), 1 affected with a suspected allopurinol-induced disease, 2 affected with another suspected drug induced disease. At each SNP, an additive model was considered and two ORs were computed to model the effect of the SNP among allopurinol-induced affected individuals (OR_allopurinol_) and among other affected individuals (OR_otherdrugs_). The null hypothesis tested was one of no effect of the SNP in any of the strata, i.e. OR_allopurinol _= OR_otherdrugs _= 1. It was tested using a 2 degrees-of-freedom chi-square test. The significance of the difference between OR_allopurinol _and OR_otherdrugs _was also tested in this model using the corresponding one degree-of-freedom chi-square test.

In the same way, a multinomial logistic model was used to determine whether genetic differences exist depending on the disease severity. A four class disease status was used: 0 unaffected (controls), 1 SJS, 2 SJS/TEN overlap and 3 TEN.

Furthermore, haplotype association was tested using Plink v1.06 [23].

For all the logistic regression modelling and haplotype association testing, an adjustment on the first two PCs obtained from the PCA of the genotype data was used.

## Results

The clinical details of the 424 patients remaining after quality-control are summarized in Table 1: 61.3% are female, 47.2% have a SJS phenotype, 34.4% a SJS/TEN overlap phenotype and 18.4% a TEN phenotype. A majority of the patients was sampled from France (110 patients) and Germany (277 patients). History of drug intake was carefully monitored and a subgroup of 57 patients (13.4%) with potentially allopurinol-induced reactions was identified. There is no difference in phenotype distribution between males and females but there are some differences depending on the drug and the country where samples were collected. There is a lack of TEN among allopurinol-induced patients (test of homogeneity of the three disease forms in allopurinol induced versus non-allopurinol induced partients: chi-square = 7.89, p-value = 0.019) and a lack of TEN among patients from Germany as compared to patients from France (chi-square = 23.68, p-value = 7.2 10-6). The two variables however are not independent since allopurinol is more used in Germany than in some other countries and in particular France.

**Table 1:** Description of the sub-sample of 424 patients selected for the analysis

Number of patients	SJS	SJS/TEN	TEN	Total
Total	200 (47.2%)	146 (34.4%)	78 (18.4%)	424
Females	124	89	47	260 (61.3%)
Allopurinol-induced	33	21	3	57 (13.4%)
From France	41	32	37	110 (25.9%)
From Germany	145	97	35	277 (65.3%)
From Other countries	14	17	6	37 (8.7%)

The number of patients (proportion of the total of 424 patients in percents) with the different phenotypes is presented in the total sample, among the female patients and as a function of the country where patients were identified.

**Figure 3:**
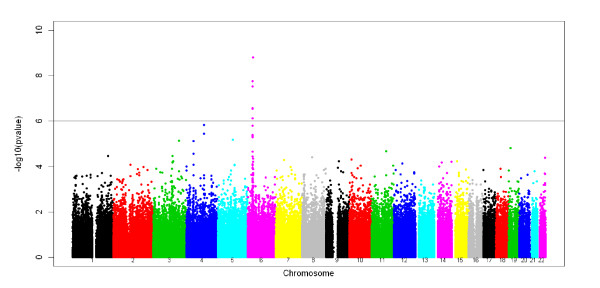
**Results of the genome-wide association screen**. The -log(p-value) of the PC-corrected Armitage-trend test implemented in Eigenstrat are plotted for the 268,818 autosomal SNPs that passed the QC with the different colors representing the different chromosome. The horizontal line represents the 10^-6 ^p-value threshold that also corresponds to a False Discovery Rate q-value of 5%.

**Figure 4:**
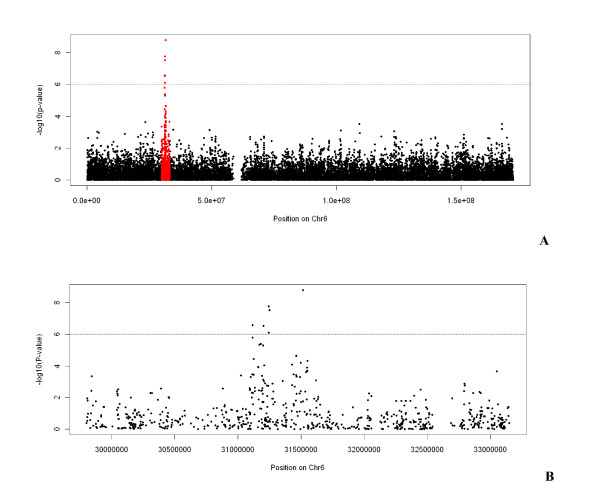
**Results of the association test on chromosome 6**. **A **The -log(p-value) of the PC-corrected Armitage-trend test implemented in Eigenstrat are plotted for the 18,1278 SNPs located on chromosome 6 as a function of their position on the chromosome in base pairs (Build 36). SNPs located in the HLA region are highlighted in red.** B **Detail of the association signals for the 706 SNPs located in the HLA region. The horizontal line represents the 10^-6 ^p-value threshold.

**Figure 5:**
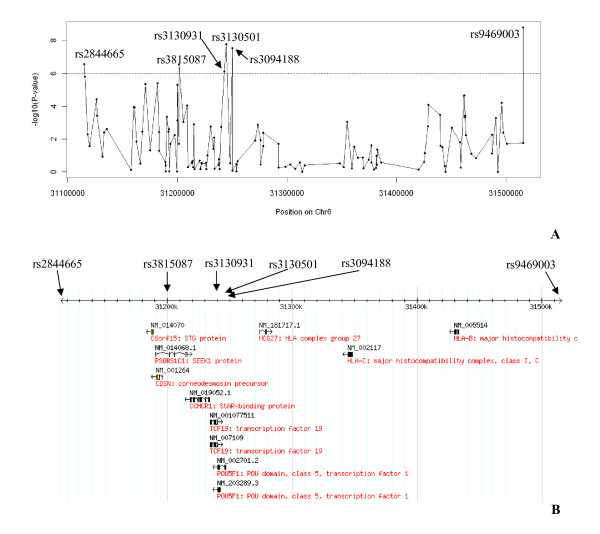
**Association results in the 401 kb region encompassing the top six signals**. **A **The -log(p-value) of the PC-corrected Armitage-trend test are plotted against the chromosome 6 positions for the 126 SNPs located in the region.** B **Listing of genes located in this region based on the HapMap web-browser.

For association testing, the 424 patients were compared to the 1,881 controls from the reference panel originating from France (1,228 individuals) and Germany (653 individuals) who were also the closest controls to the cases on the PCA plot (Figure 1). We then tested each SNP for association controlling on the first two PCs of the PCA using Eigenstrat [24]. Results are presented in Figure 3. One genome-wide significant signal was detected in the MHC region on chromosome 6 where 6 SNPs showed False Discovery Rate adjusted q-values below 5% (see Figure 4 and Table 2). The most significant SNP, rs9469003, is located at position 31,515,807, ~85 kb upstream of the HLA-B locus and has an OR of 1.73 (95%CI = [1.44;2.08]). The five other SNPs are all located in a more telomeric region, 250 kb apart from rs9469003, in between positions 31,114,834 and 31,250,224 (see Figure 5). There is very limited Linkage Disequilibrium (LD) between these five SNPs and the top one and the six SNPs defined 13 haplotypes with a frequency of 1% or above. The haplotype CACGAC formed by the risk allele at each locus showed the strongest association with the disease with an OR of 2.84 (95%CI = [2.03; 3.98]) that is significantly higher than the one observed for rs9469003 only. To dissect further this haplotypic association, the haplotype conditioning test implemented in Plink v1.06 was used and we found that none of the 6 SNPs by itself has an independent effect nor explains the haplotype association.

**Table 2:** List of most significant SNPs associated with SJS/TEN

SNP	Chr	Position Build 36	RiskAllele (Other Allele)	Risk Allele Frequency in Cases	Risk Allele Frequency in Controls	PC corrected Trend test^b^	p-value^b^	q-value^c^	OddsRatio^d^	95% CI^d^	Pvalue of HW test	Annotation^a^
rs2844665	6	31114834	C (T)	0.72	0.62	26.46	2.69 10^-7^	1.48 10^-2^	1.54	1.30-1.82	0.77	C6orf205 flanking_3UTR
rs3815087	6	31201566	A (G)	0.31	0.21	26.32	2.89 10^-7^	1.54 10^-2^	1.53	1.29-1.80	0.08	PSORS1C1 UTR
rs3130931	6	31242867	C (T)	0.77	0.69	24.41	7.78 10^-7^	3.34 10^-2^	1.54	1.29-1.84	0.66	POU5F1 Intron
rs3130501	6	31244432	G (A)	0.83	0.74	31.78	1.73 10^-8^	0.22 10^-2^	1.74	1.43-2.13	0.59	POU5F1 Intron
rs3094188	6	31250224	A (C)	0.72	0.63	30.75	2.93 10^-8^	0.26 10^-2^	1.59	1.34-1.88	0.06	POU5F1 Flanking_5UTR
rs9469003	6	31515807	C (T)	0.24	0.15	36.41	1.60 10^-9^	0.04 10^-2^	1.73	1.44-2.08	0.003	HCP5 flanking_5UTR

**^a ^**Annotations were obtained from the Illumina annotation file.

**^b ^**Results obtained using the test implemented in Eigenstrat

**^c ^**q-values (FDR adjusted p-values) were obtained by using the R package fdrtool [50].

**^d ^**Results obtained using plink logistic regression test accounting for the top 2 PC.

Using a multinomial logistic regression approach, we found that the association with haplotype CACGAC was much stronger in the subgroup of patients with an allopurinol induced disease (ORallopurinol = 7.77, 95%CI = [4.66; 12.98]) than in other patients (ORotherdrugs = 1.92, 95%CI = [1.40;2.64]). The equality of odds-ratio (ORallopurinol = ORotherdrugs) was strongly rejected (p = 6.56 × 10^-7^). Results were the same in France and Germany a shown in Additional File 1 Tables S1 and S2.

To determine how much of the observed signal could be explained by the known association between allopurinol-induced SJS/TEN and HLA-B*5801, we took advantage of the availability of HLA-B two-digit resolution genotypes for 74 of the 424 patients [16]. Among the 74 HLA-B genotyped patients, 11 were carriers of an HLA-B58 allele and had an allopurinol-induced disease. The frequency of the CACGAC haplotype is increased in HLA-B58 carriers as compared to non HLA-B58 carriers (32.44% versus 10.44%, p-value of the test adjusted on the top 2 PC = 0.0052) but the linkage disequilibrium is not complete. Note however that given the small sample size of HLA-B typed individuals, the power was quite low to detect this difference (power of 60% at a type-one error rate level of 5%).

## Discussion

Discovering genes involved in severe cutaneous adverse reactions and especially SJS/TEN is a major challenge for pharmacogenetics as these reactions, when not fatal, are a sword of Damocles for those who already had the disease and fear to take any drug. Despite important efforts, only genes located in the HLA region have been identified so far. One possible explanation for this lack of success is the limited sample sizes of patients available. Indeed SJS/TEN is fortunately a very rare disease and in most studies, sample sizes rarely exceed one or two hundreds of patients, making it difficult to investigate more than a few candidate genes. Through a collaborative effort, the RegiSCAR group was able to collect detailed medical information and DNA of more than half a thousand of patients from Europe who were genotyped on Illumina 317 K chips. By comparing their genotypes to the ones of 1,881 controls genetically matched for the country of origin, we were able to study the SJS/TEN association at a genome-wide level on European samples. Apart from six SNPs located in the HLA region, no other locus was found to be associated with the disease at a high enough significance level to ensure it is not false positive due to the multiple tests performed. Sample sizes of half a thousand are not enough to ensure a good power to detect common variants with small effects (OR in the range between 1.1 and 1.3) similar to those identified in several common diseases such as diabetes or different cancers [29] where larger samples are easier to collect. However, given the 424 patients remaining after Quality Control, the study was powerful enough to detect common variants with modest effects (the power exceeded 80% to detect variants with an allele frequency above 15% conferring ORs above 1.7 under a multiplicative model). The fact that only the HLA region is detected, suggests that there might not be any other common variant that confer a substantial increase in disease risk and could thus be of interest as a predictive factor. These power computations might however be too optimistic as they are based on the assumption that the same genetic variants might be involved in cases of SJS/TEN associated to different drugs. If we consider that there might exist some levels of genetic heterogeneity depending on the drugs involved as it is the case for HLA associations, the power can be dramatically reduced. For example, considering only the 57 allopurinol-exposed cases, the power to detect a variant of frequency 15% conferring an OR of 2 is then only 5% at a nominal type-one error rate value of 10^-6^. On the other hand, the power to detect a similar effect than the one observed at rs9469003 (i.e., risk allele of frequency of 15% with an associated OR of ~4 among allopurinol-induced SJS/TEN) is 99%, showing that the study was not underpowered to detect strong effects even if they were restricted to a small subset of patients.

It is true however that we might have missed some variants with important effects, especially those with minor allele frequencies below 5% that are not well covered by SNP-chips and it could thus be of interest to investigate the association with rare variants. If the "common disease-common variant hypothesis" was believed to explain the susceptibility to multifactorial diseases [30,31], results from GWAS have shown that common variants only explain a minor part of most common disease heritabilities. It has been suggested that rare variants could explain at least part of this "missing heritability" and indeed, rare variants have been found in several diseases [32-36]. Interestingly these rare variants are often functional variants with a direct impact on the protein functionality and they usually confer a much stronger increase in disease risk than common variants. They are also more likely to be affected by some moderate levels of negative selection [37,38]. For diseases such as adverse drug reactions, it is not unlikely that the genes and variants involved could be under selection. Indeed if most of the drugs have been introduced too recently to be directly responsible for selective pressures, they are often derived from nutrients that have been consumed by humans for a long time. Thus, genes involved in drug metabolism can potentially be involved in natural selection and this was confirmed by previous studies [39-41]. However, the implication of drug metabolism genes in SJS/TEN remains to be established.

Concerning the association with HLA detected in this sample, we confirmed that it is drug-specific with the strongest association found for the group of patients where allopurinol is suspected to be the cause of the disease. However the disease association was still detectable after exclusion of all patients exposed to allopurinol (whether considered as causing the reaction or not) (OR = 2.13, 95% CI = [1.41; 3.23]). This suggests that different HLA alleles are probably involved depending on the drug, and the 6 SNPs identified could be those that were in linkage disequilibrium (LD) with several of these HLA alleles. To further explore this hypothesis, we tried to impute HLA-B genotypes from the SNP data [42] but we were not successful because of the presence of multiple rare alleles at this locus such as HLA-B58 itself that is only present in one of the European families genotyped by de Bakker et al. [43] precluding the possibility to find appropriate tags for this allele using this sample. To overcome the problem, we tried to determine tag SNPs using our own HLA-B genotyped sample that was enriched in HLA-B58 but we did not find any SNP or group of SNPs that was correlated enough with the HLA-B58 allele (the maximum r^2 ^value was below 20%). This poor HLA-B tagging ability of the SNPs could certainly explain why the association found here in relation with allopurinol is much weaker than the one reported in studies on European samples using resolved HLA-B alleles [16,44]. However, even in these two latter studies, the association with HLA-B*5801 is much weaker than the one found in Han Chinese [15] or Thai populations [45] where all patients with allopurinol-induced SJS/TEN are carriers of this allele. It is thus possible that the HLA genetic determinants involved in SJS/TEN are not the HLA-B alleles themselves but some loci in LD with them. Further studies in this genomic region would certainly be necessary to better understand the mechanisms involved.

On a methodological point of view, this study illustrates how Reference Control Panels could be used to test for association when only cases are genotyped. Only patients were genotyped here and controls were selected from the CNG European Control Panel [22] based on their country of origin that was either France and Germany, since those were the two main countries where the cases come from. We also tried to use the whole panel of controls and correct for population stratification using Principal Component Analysis (PCA) and an adjustment on up to 20 PCs instead of the 2 used here. We found that including controls that were more genetically distant to the cases does not really improve the signal in the HLA region and instead could lead to false positive results in other genomic regions. This is somewhat in contradiction with the results recently reported by Zhuang et al. [46] and will probably need to be further investigated. We also used some alternative approaches where controls were genetically matched to cases using either a distance based on the identity by state as described by Guan et al. [47] or a distance derived from the top PCs of the PCA performed to identify axes of variations similar to the one proposed by Luca et al. [48]. Tests accounting for the matching units were then performed and we found that only the HLA region pointed out with significance levels in the same order of magnitude as the ones obtained here. These matching strategies might however be of interest as they could allow the analysis of more heterogeneous samples by including for example those non-European RegiSCAR cases that were excluded here and matching them to individuals from the CEPH-Human Genome Diversity Panel [49].

## Conclusion

Our study confirms the involvement of genetic variants located in the HLA region in the susceptibility to SJS/TEN in European samples, especially in association with allopurinol. No difference is seen depending on the disease severity and no other locus reaches genome-wide association in this sample that is also the largest one collected so far.

## Competing interests

The authors declare that they have no competing interests.

## Authors' contributions

MM, MS, JCR, AH, ML designed the study, DZ, ML supervised the genotyping, LN, YL, JCR, MM, PS were responsible for clinical data collection, RK, EG performed the statistical analysis, EG wrote the manuscript and all authors contributed to and approved the final draft.

## Supplementary Material

Additional file 1 **Supporting material for manuscript "Genome-Wide Association study of Stevens-Johnson Syndrome and Toxic Epidermal Necrolysis in Europe" Results of association tests for the top SNP (Table S1) and for the most associated haplotype (Table S2) after stratification on the country of origin**. Click here for file
