# Supplementary material for: Genome-wide association study of Stevens-Johnson Syndrome and Toxic Epidermal Necrolysis in Europe
Source: Orphanet J Rare Dis. 2011 Jul 29;6:52. doi: 10.1186/1750-1172-6-52 (PMC3173287; doi:10.1186/1750-1172-6-52)
Supplement: Additional file 1 — Supporting material for manuscript "Genome-Wide Association study of Stevens-Johnson Syndrome and Toxic Epidermal Necrolysis in Europe" Results of association tests for the top SNP (Table S1) and for the most associated haplotype (Table S2) after stratification on the country of origin. [file 1750-1172-6-52-S1.DOC]

|  | **Cases^b^** | **Controls^c^** | **OR [95% CI]^d^** |
| --- | --- | --- | --- |
| **All patients (424^a^) versus All controls (1870^a^)** | 0.24 (207) | 0.15 (549) | 2.84 [2.03; 3.98]^e^ |
| **All patients exposed to allopurinol (57^a^) versus All controls (1870^a^)** | 0.46 (52) | 0.15 (549) | 4.04 [2.74; 5.97]^e^ |
| **French patients (4^a^) exposed to allopurinol versus French Controls (1218^a^)** | 0.75 (6) | 0.14 (360) | 17.30 [3.48; 86.05]^f^ |
| **German patients (43^a^) exposed to allopurinol versus German Controls(652^a^)** | 0.41 (35) | 0.15 (189) | 4.05 [2.56; 6.39]^f^ |

**Table S1** Association at the top SNP rs9469003 depending on the country of origin and the drug**.**

**^a^** Number of individuals genotyped at the marker

**^b^** Frequency of the C allele in cases (number of alleles)

**^c^** Frequency of the C allele in controls (number of alleles)

**^d^** Odds-Ratio under a multiplicative model

**^e^** These ORs were adjusted on the first two PCs to account for population stratification

**^f^** Woolf’s test of heterogeneity between these two OR (France and Germany) is not significant (chi-square (1df)=2.91, p-value=0.088)

|  | **Cases^a^** | **Controls^b^** | **OR [95% CI]^c^** |
| --- | --- | --- | --- |
| **All patients versus All controls** | 9.45 | 4.01 | 2.84 [2.03; 3.98] |
| **All patients exposed to allopurinol  versus All controls** | 24.16 | 4.01 | 7.77 [4.66; 12.98] |
| **French patients exposed to allopurinol versus French Controls** | 33.83 | 4.15 | 11.23 [1.23; 107.00] |
| **German patients exposed to allopurinol versus German Controls** | 11.11 | 3.61 | 9.32 [4.24; 20.50] |

**Table S2** Association with the CACGAC haplotype depending on the country of origin.

**^a^** Frequency (%) of the haplotype in cases

**^b^** Frequency (%) of the haplotype in controls

**^c^** Odds-Ratio associated to the CACGAC haplotype when the reference haplotype is CATGAC
